# Safety and Tolerability of an Antimalarial Herbal Remedy in Healthy Volunteers: An Open-Label, Single-Arm, Dose-Escalation Study on *Maytenus senegalensis* in Tanzania

**DOI:** 10.3390/tropicalmed7120396

**Published:** 2022-11-25

**Authors:** Kamaka Kassimu, Florence Milando, Justin Omolo, Abel Mdemu, Gloria Nyaulingo, Hussein Mbarak, Latipha Mohamed, Ramla Rashid, Saumu Ahmed, Mohammed Rashid, Hania Msami, David Damiano, Beatus Simon, Thabit Mbaga, Fatuma Issa, Omar Lweno, Neema Balige, Omary Hassan, Bakari Mwalimu, Ali Hamad, Ally Olotu, Andreas Mårtensson, Francis Machumi, Said Jongo, Billy Ngasala, Salim Abdulla

**Affiliations:** 1 Bagamoyo Clinical Trial Facility, Ifakara Health Institute, Bagamoyo P.O. Box 74, Tanzania; 2 Department of Parasitology and Medical Entomology, Muhimbili University of Health and Allied Sciences, Dar es Salaam P.O. Box 65001, Tanzania; 3 Department of Traditional Medicine, National Institute for Medical Research, Dar es Salaam P.O. Box 9653, Tanzania; 4 Department of Women’s and Children’s Health, International Maternal and Child Health (IMCH), Uppsala University, S-751 85 Uppsala, Sweden; 5 Institute of Traditional Medicine, Muhimbili University of Health and Allied Sciences, Dar es Salaam P.O. Box 65001, Tanzania

**Keywords:** safety, tolerability, antimalarial, herbal remedy, healthy volunteer, *M*. *senegalensis*, Tanzania

## Abstract

Background: Though *Maytenus senegalensis* is one of the medicinal plants widely used in traditional medicine to treat infectious and inflammatory diseases in Africa, there is a lack of safety data regarding its use. Therefore, the study aimed to asselss the safety and tolerability of the antimalarial herbal remedy *M. senegalensis*. Material and Methods: The study design was an open-label, single-arm, dose-escalation. Twelve eligible male healthy Tanzanians aged 18 to 45 years were enrolled in four study dose groups. Volunteers’ safety and tolerability post-investigational-product administration were monitored on days 0 to 7,14, and 56. Results: There were no deaths or serious adverse events in any of the study groups, nor any adverse events that resulted in premature discontinuation. The significant mean changes observed in WBC (*p* = 0.003), Neutrophils (*p* = 0.02), Lymphocytes (*p* = 0.001), Eosinophils (*p* = 0.009), Alanine aminotransferase (*p* = 0.002), Creatinine (*p* = 0.03) and Total bilirubin (*p* = 0.004) laboratory parameters were not associated with any signs of toxicity or clinical symptoms. Conclusions: *M. senegalensis* was demonstrated to be safe and tolerable when administered at a dose of 800 mg every eight hours a day for four days. This study design may be adapted to evaluate other herbal remedies.

## 1. Introduction

*Maytenus senegalensis* (Lam.) Exell is one of the medicinal plants belonging to the Celastraceae family. It is a shrub or tree that grows up to 15 m high and is widely distributed in most African countries as well as Arabia, Afghanistan and India [1]. The different parts of the plant are widely used in traditional medicine for the treatment of infectious and inflammatory diseases in Africa. The roots of *M. senegalensis* contain several active substances, such as triterpenes, sterols, saponosids, alkaloids and tannins [1,2,3]. 

Pharmacological assays have confirmed that most of these substances have biological activities. *M. senegalensis* is reported to be anti-inflammatory [1,3], antimalarial, analgesic [2,4,5], antitussive [6], antibacterial and anthelminthic [7,8,9,10,11]. In Tanzania, *M. senegalensis* is one of the most important medicinal plants for the treatment of malaria, fever, pain and chronic diseases [12]. Traditional uses of *M. senegalensis* have also been reported from other African countries, such as Benin, Ivory Coast, Kenya, Sudan, Zambia and Zimbabwe [7,13,14]. 

Studies have been conducted to determine the acute toxicity of *M. senegalensis* ethanol extract at different doses in mice. The *M. senegalensis* extract was found to be nontoxic, and the oral median lethal dose in mice was determined to be greater than 1600 mg/kg body weight [4]. In another study, the extract of *M. senegalensis* at a dose of 5000 mg/kg body weight was well tolerated, without causing visible signs of toxicity or mortality in mice [15]. Thus, *M. senegalensis* ethanol extract can be categorized as a nontoxic crude drug that acts harmlessly under normal traditional usage without causing acute toxic symptoms and complications.

There is a claim that products with a long history of popular use are generally safe when used properly at common therapeutic doses [16]. The lack of evidence of side effects in the context of traditional herbal medicine use does not rule out the possibility of their occurring. Drug safety is established through properly designed phase 1 and 2 clinical trials. Therefore, the lack of safety data for an antimalarial herbal remedy *M. senegalensis* obtained from clinical trials necessitated the need to conduct this study. 

This study was conducted to evaluate the safety and tolerability of antimalarial herbal remedy *M. senegalensis* in healthy Tanzanian adult volunteers. This study may demonstrate a new development strategy for identifying novel antimalarial drugs that can counteract the emergence of antimalarial resistance through exploiting the rich natural biodiversity of Africa to accelerate malaria elimination [17,18,19]. Moreover, clinical evaluation of the medicinal potential of herbal products may fast track finding solutions for malaria and other health problems in Africa.

## 2. Materials and Methods

### 2.1. Study Design

The study design was an open-label, single-arm, dose-escalation study to evaluate the safety and tolerability of antimalarial herbal remedy *M. senegalensis* in healthy adult Tanzanian volunteers (ClinicalTrials.gov Identifier: NCT04944966. Accessed on 14 November 2022). 

**Figure 1:**
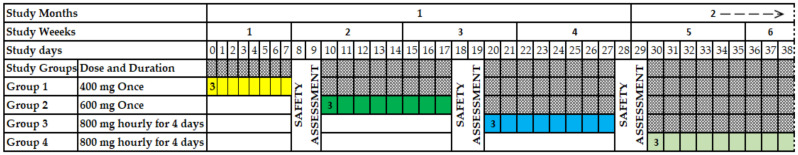
Study design. The yellow color presents the safety assessment days for group 1; the green color presents the safety assessment days for group 2; the light blue color presents the safety assessment days for group 3; and the light green color presents the safety assessment days for group 4.

The study volunteers were divided into four groups (1, 2, 3 and 4), with three volunteers in each group. The volunteers from group 1 were enrolled first in the study, given 400 mg of *M. senegalensis* and followed by a safety evaluation for seven days after the administration. The volunteers of group 2 were enrolled next in the study and given 600 mg of *M. senegalensis* and followed by a safety assessment for seven days after administration. Then, the volunteers from group 3 were enrolled in the study and given 800 mg of *M. senegalensis* every 8 h for 4 days, followed by a safety evaluation for 7 days after the first administration. Lastly, volunteers of group 4 were enrolled in the study and given 800 mg of *M. senegalensis* every 8 h for 4 days (Figure 1). Study volunteers from groups 3 and 4 were given an 8 hourly dose of 800 mg *M. senegalensis* to generate safety data for the highest dose from the six volunteers.

All medications other than the tested product were recorded as concomitant medications. Neither routine activities nor dieting were restricted during the trial period.

All study volunteers were hospitalized for 7 days. The dose of *M. senegalensis* was administered under direct observed treatment (DOT). As a precaution, women were not included in the study to reduce the risk of reprotoxicity, because preclinical data on reproduction toxicity were not available.

The trial was carried out from June to September 2021 at the Bagamoyo Clinical Trial Facility (BCTF), located on the Kingani Estate, 74 km north of Dar es Salaam within the coastal region.

### 2.2. Study Population

The study population included healthy men aged 18 to 45 years with a Body Mass Index (BMI) ranging from 18 to 30 kg/m^2^ and who were long-term residents of the study area and agreed to release medical information and to be attended by a study clinician prior to and during the study. Other inclusion criteria were the volunteer’s agreement to provide contact information for a third-party household member or close friend to the study team, available by mobile phone 24 h during the study period, agreement not to participate in another clinical trial and donate blood during the study period, health that complied with the study protocol procedures and agreement to undergo HIV, hepatitis B (HBV) and hepatitis C (HCV) tests, demonstrated understanding of the study, signed written informed consent and free of malaria parasitemia by blood smear at enrolment. 

Volunteers were excluded if they had any of the following criteria: received an investigational malaria drug in the last 5 years, participation in any other clinical study involving investigational medicinal products within 30 days prior to the onset of the study, history of arrhythmias, prolonged QT-interval or another cardiac disease, clinically significant abnormalities in electrocardiogram (ECG) at screening, a positive family history of cardiac disease in a first- or second-degree relative at the age of 50, a history of psychiatric disease, suffering from any chronic illness (diabetes mellitus, cancer, or HIV/AIDS), any confirmed or suspected immunosuppressive or immune-deficient condition, history of drug or alcohol abuse, use of chronic immunosuppressive drugs or other immune-modifying drugs within three months of study onset except inhaled and topical corticosteroids. Other exclusion criteria were any clinically significant deviation from the normal range in biochemistry or hematology, blood tests or urine analysis; positive HIV, hepatitis B virus or hepatitis C virus tests; suspicion of having clinically active TB history or physical examination with a positive QuantiFERON-TB Gold Test in-tube assay; symptoms, physical signs, and laboratory values suggestive of systemic disorders that could muddle the interpretation of the study results or jeopardize the health of volunteers. The last exclusion criterion was any medical, social or occupational reason that, in the judgment of the study clinician, was a contraindication to participation or impaired the ability to give informed consent, increased the risk to the volunteer due to participation in the study, affected the ability of the volunteer to participate in the study or impaired interpretation of the study data. 

### 2.3. Sample Size

The study aimed to assess the safety and tolerability of an antimalarial herbal remedy. Therefore, the calculation of statistical power and sample size was not performed. The sample size for the study was set at 12 volunteers. The study was carried out using adapted dose escalation rules for the traditional 3 + 3 design, which is commonly used in oncology phase 1 clinical trials [20]. The regulatory justification for the minimum sample size for the phase 1 clinical trial on the effect of food on the bioavailability of investigational products was used as a justification for the sample size of 12 healthy volunteers [21].

### 2.4. Preparation of M. senegalensis 

The root barks of *M. senegalensis* were harvested on 9 June 2020 from Bugabo buzi village, Misenye District in the Kagera Region. The region has two rain seasons: heavy rain from March to May and short rains from September to December. The plant was identified by Haji O. Selemani, a botanist from the University of Dar es Salaam, with Voucher Specimen Number 5672, which was deposited at the University Herbarium. The root barks were then debarked to obtain the root barks, which were air-dried under shade at room temperature for two weeks and then pulverized by a grinding mill. The pulverized root backs (20 kg) were extracted three times by cold percolation at room temperature using 80% ethanol and filtered by Macherey–Nagel filter paper. The filtrates were distilled under reduced pressure at 40 °C to give 2.24 kg of brown gummy extract.

**Figure 2:**
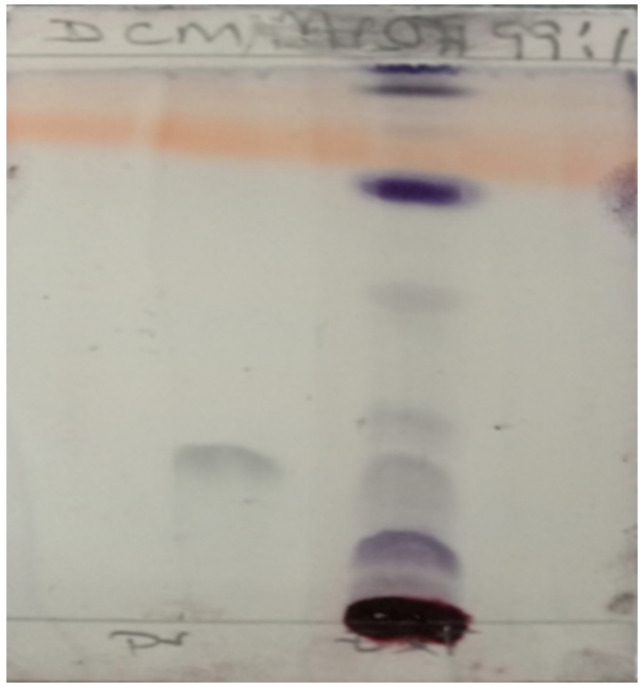
Thin layer chromatography (TLC) shows the presence of an active ingredient (Pristimerin).

The presence of an active ingredient (Pristimerin) for the treatment of malaria was qualitatively determined using the Thin Layer Chromatography (TLC) method. The extract (10 mg) and Pristimerin (1 mg) were separately dissolved in methanol (1 mL). The solutions were then spotted on a TLC and eluted with dichloromethane/methanol at 99:1. The Pristimerin in the sample was observed at Rf 0.3, matching the standard Pristimerin (Figure 2). 

**Figure 3:**
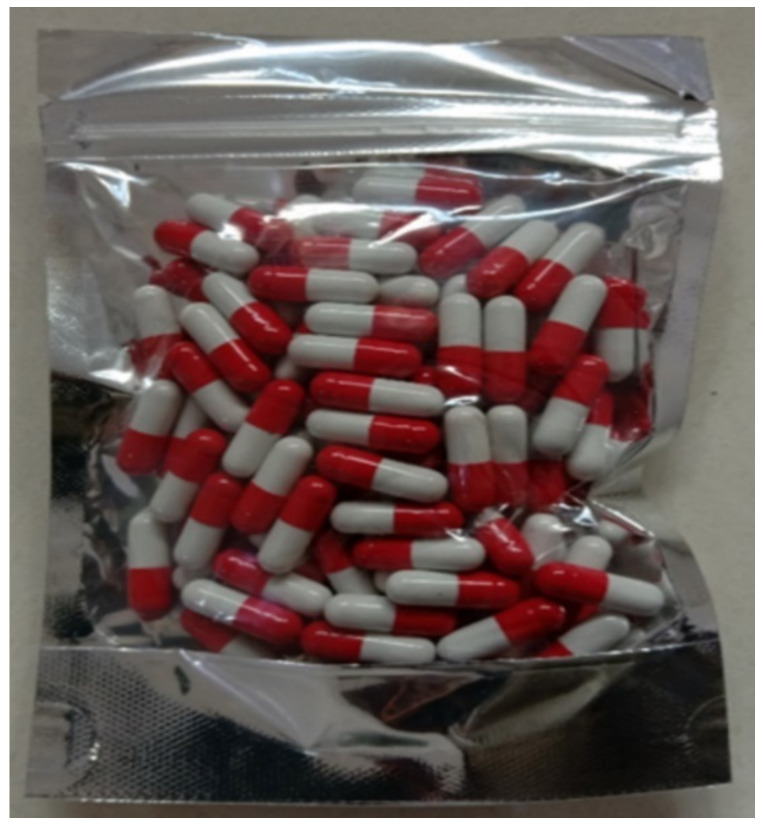
Blister bag containing 100 capsules.

The extract was granulated by mixing it with starch at a ratio of 2:1 (Extract 2: Starch 1) and packed in hard shell capsules. Each capsule contained 300 mg of powder (200 mg of extract and 100 mg of starch). These capsules were packed in blister bags containing 100 capsules (Figure 3).

A trial batch of investigational products was analyzed at the Institute of Traditional Medicine-MUHAS, Tanzania Bureau Standards & Government Chemist Laboratory Authority (GCLA) to minimize the risks of misidentification [22]. The test results revealed that the trial batch had met the standards of Tanzania (TZS 2155:2018). Furthermore, no aflatoxins were detected in the trial batch sample submitted to the Tanzania Bureau of Standards (TBS) for analysis. Refer to Table 1.

The balance between efficacy and safety was considered during the selection of a dose for the estimation of the human equivalent dose (HED). The toxicity of drugs increases with increasing doses. According to a previous study, a dose of 100 mg/kg body weight was safe and was able to clear parasites by almost 100%; it was also demonstrated that it was safe at doses as high as 1600 mg/kg body weight [4]. Therefore, the no observed adverse effect level (NOAEL) dose during estimation of the maximum recommended starting dose (MRSD) in humans was 1600 mg/kg body weight. Hence, the MRSD in humans extrapolated from the animal NOAEL dose is 7800 mg/10 = 780 mg, or 800 mg. The US FDA guideline for the estimation of the maximum safe starting dose guided the extrapolation of the animal dose to a human dose [23].

### 2.5. Safety Assessment

The safety of the tested product was evaluated through taking medical history, physical examination, vital signs, 12-lead ECG, clinical laboratory tests and incidence of AEs. Physical examinations were performed by study clinicians and included the examination of the following: general appearance, eyes, ears, nose, throat, chest/respiratory, heart/cardiovascular, gastrointestinal/liver, musculoskeletal/extremities, dermatological/skin, thyroid/neck, lymph nodes, and neurological/psychiatric systems.

The vital signs observed during the study included systolic blood pressure, diastolic blood pressure, pulse rate, and body temperature at baseline and 0, 1, 2, 3, 4, 5, 6, 7, 14, 28 and 56 days. A 12-lead ECG was performed at baseline and at 3, 7, 14, 28 and 56 days.

Safety laboratory tests (including hematology and chemistry) were performed at baseline and at 3, 7, 14 and 28 days. A total of 1 mL of venous blood sample was collected into a K3 EDTA vacutainer tube (Greiner Bio-one) and a Z-serum clot activator tube (Greiner Bio-one) for hematology and biochemistry analysis, respectively. For hematological analysis, a fully automated 5-part differential hematology analyzer (Sysmex XS-800i, Sysmex Corporation, Japan) was used to analyze samples within 8 h of blood draw. The results printed out were double-checked by two independent, qualified technicians before being handed out to clinicians for clinical management. For biochemical analysis, samples were allowed to stand at room temperature for 30 min before separating the serum by centrifugation at 3000 rpm for 10 min. The serum was analyzed using Cobas Integra 400 Plus (Roche Diagnostic, Switzerland) within 24 h after the blood draw. If the testing was to be delayed, serum for biochemical analyses was stored at −20 °C and subjected to a single freeze–thaw cycle at the time of analysis, and whole EDTA blood for hematological analyses was stored at 4–8 °C and analyzed within 24 h after the blood draw.

For the quality assurance of the analyzers, internal quality control (IQC) and external quality assurance (EQA) quality assessments were performed on the main and back-up analyzers according to standard operating procedures. IQC was carried out daily using quality control materials supplied by the respective analyzer’s manufacturer. EQA was performed monthly using materials supplied by an independent EQA firm (the College of American Pathologists (CAP) for the biochemistry analyzer and the United Kingdom National External Quality Assessment Scheme (UK NEQAS) for the hematology analyzer). Only the analyzers that passed the IQC and EQA assessments were used for clinical sample analysis.

White blood cell count (3.48 to 9.11 × 10^3^/L), red blood cell count (4.39 to 6.5 × 106/L), hemoglobin (12.6 to 17.3 g/dL), platelets (107 to 396.2 × 10^3^/L), neutrophil counts (1.18 to 5.46 × 10^3^/L), lymphocyte counts (1.19 to 3.4 × 10^3^/L), and eosinophil counts (0 to 0.78 × 10^3^/L) were all examined. The analysis of liver function tests included AST (12.3 to 74.7 U/L), ALT (3.5 to 46.8 U/L) and total bilirubin (3.1–31.1μmol/L). The serum creatinine level (49 to 95.3 μmol/L) was measured as a function of the kidney.

These laboratory reference intervals were derived from a local or regional population that was more likely to have similar biological characteristics, as stipulated in the Clinical and Laboratory Standards Institute (CLSI) guidelines [24]. The correct interpretation of laboratory results requires accurate clinical laboratory reference intervals derived from a local or regional population [25].

Solicited adverse events were monitored and recorded up to day 7, while unsolicited adverse events were monitored and recorded up to day 28 after the first administration of the tested product.

All study volunteers were monitored throughout the study for serious adverse events (SAEs). The study clinicians were responsible for evaluating all AEs in terms of intensity (mild, moderate, or severe), duration, severity, outcome, and relationship to the study drug. The Standard Treatment Guidelines & National Essential Medicines List, Tanzania Mainland (STG/NEMLIT-2017); Division of AIDS (DAIDS) Table for Grading the Severity of Adult and Pediatric Adverse Events (Corrected Version 2.1, July 2017); US FDA, Toxicity Grading Scale for Healthy Adult and Adolescent Volunteers Enrolled in Preventive Vaccine Clinical Trials: Guidance for Industry (September 2007); and Common Terminology Criteria for Adverse Events (CTCAE) (Version 5.0, November 2017) were used to develop the grading criteria for adverse events.

### 2.6. Data Analysis

Case report forms were created to collect clinical and biological data for each volunteer. Data were first recorded on a paper CRF and then transcribed into the Castor Electronic Data Capture (EDC) system and saved in an electronic case report form (eCRF). Furthermore, data from the castor EDC were extracted and transferred to Stata Statistical Software version 15 for further analysis. Means and standard deviations were reported for continuous numbers and percentages for categorical variables. The comparison of the different laboratory parameter means over time was done using Friedman’s test. A *p*-value ≤ 0.05 was considered significant.

## 3. Results

### 3.1. Study Volunteer Disposition 

**Figure 4:**
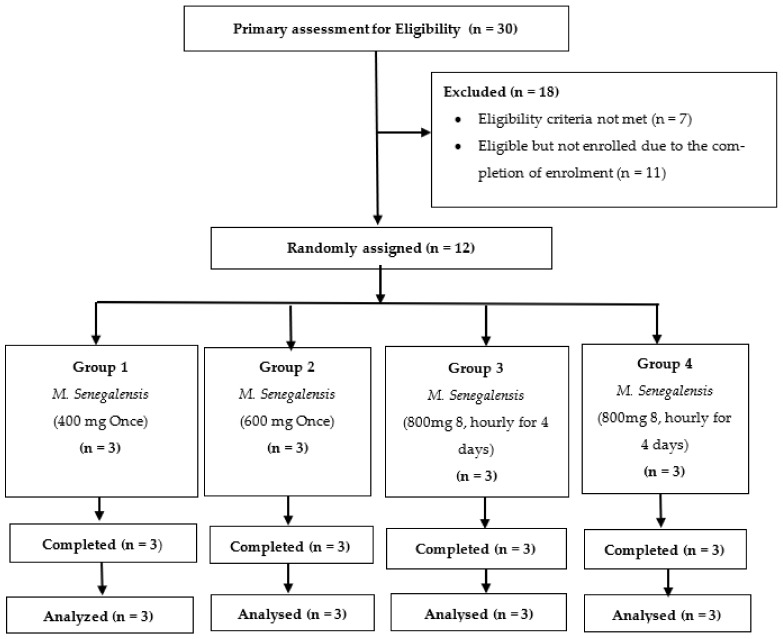
Trial flow chart showing volunteer disposition.

Only 12 of the 30 study volunteers who had been screened were eligible for randomization. These 12 eligible volunteers were randomized to four study groups (1, 2, 3 and 4), with three (3) volunteers in each study group. The 12 enrolled volunteers completed the study and were included in the analysis. Figure 4 summarizes information about the study volunteers.

### 3.2. Demographic Characteristics

The 12 study volunteers enrolled in the study were African males with a mean age of 27.70 ± 6.41 years. The average body mass index (BMI) of these volunteers was 22.77 ± 2.87 kg/m^2^. Table 2 shows the demographic characteristics of each study group.

### 3.3. Adverse Events

There were no deaths or serious adverse events in any of the study groups or adverse events that led to premature discontinuation of the study volunteers. Furthermore, no solicited or unsolicited clinical adverse events (AEs) were reported in the first 28 days of groups 1, 2, 3 and 4. All reported events were laboratory adverse events. Table 3 shows the solicited systemic adverse events in the first seven days after administration of the test products. 

**Figure 5:**
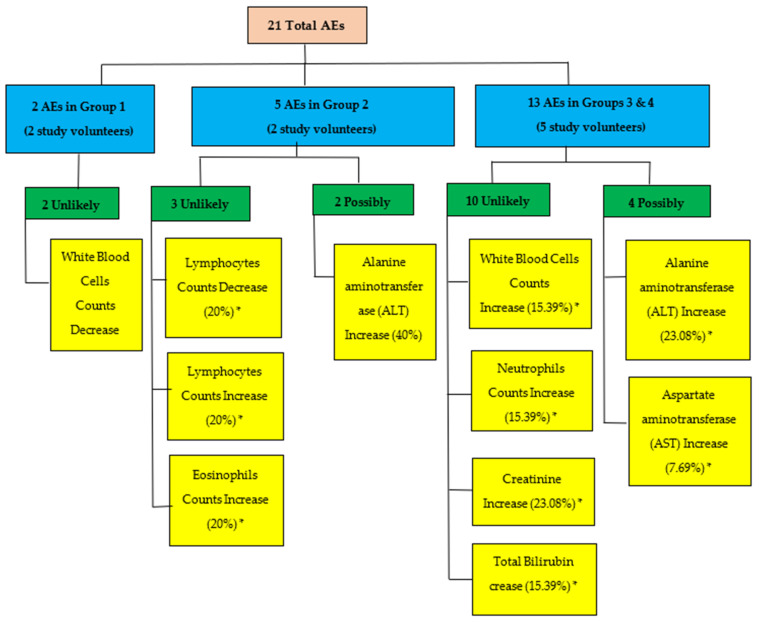
Flow diagram summarizing all adverse events (AEs) in study treatment groups based on their relationship. * Percentages are based on the number of AEs reported for each group.

A total of 21 AEs were reported throughout the study. According to Figure 5, nine (9) out of twelve (12) volunteers of study groups 1, 2, 3 and 4 experienced one or more AEs in the first 28 days. All AEs were transient and did not reach grade 3 severity. Six out of 21 (28.57%) reported adverse events were possibly related to the tested product. The causality category of the World Health Organization-Uppsala Monitoring Centre (WHO-UMC) was used to perform causality assessment.

In addition, laboratory data, vital signs, physical findings, and 12-lead electrocardiogram parameters did not show any relevant changes over time that might be related to the study drug. Clinical review of the laboratory, 12-lead electrocardiogram, vital signs, and physical examination data did not indicate any safety concerns.

### 3.4. Laboratory Test Results (Biochemistry and Hematology Responses)

Table 4 summarizes the hematology parameter mean, median, and range from inclusion to day 28 for study groups 1, 2, 3 and 4. There was a defined increasing trend in the mean value of white blood cells (WBC) for groups 2, 3 and 4. The mean value increased up to day seven and then started to return to baseline. These increasing changes are statistically significant (*p* = 0.05 for group 2 and *p* = 0.003 for groups 3 and 4). However, the highest mean value observed was within the normal range.

Furthermore, there were statistically significant changes in the mean value of platelets (PLT) for group 1 (*p* = 0.05), neutrophil count (NEUT-N) for group 3 (*p* = 0.02), lymphocyte count (LYMP_N) for group 3 (*p* = 0.001) and eosinophils count (EOS_N) for group 3 (*p* = 0.009). However, the observed changes were within the normal range.

Table 5 summarizes the mean, median and range of biochemistry parameters from inclusion until day 28 for study groups 1, 2, 3 and 4. There was a defined increasing trend in the alanine aminotransferase (ALT) mean value for groups 2, 3 and 4. The mean value increased up to day seven and then started to return to baseline. These increasing changes are statistically significant (*p* = 0.03 for group 2 and *p* = 0.002 for groups 2, 3 and 4). The highest mean value of alanine aminotransferase observed on day seven was slightly out of the normal range. However, the mean value of alanine aminotransferase on day 14 was within the normal range.

### 3.5. Laboratory Test Results (Biochemistry and Hematology Responses)

There were statistically significant changes in the mean value for creatinine (*p* = 0.03) for groups 3 and 4, which were slightly outside of the normal range for creatinine. Furthermore, there was a decreasing trend in the mean value of total bilirubin from the baseline for groups 3 and 4. The mean value of total bilirubin decreased up to day three and then started to return to baseline. These decreasing changes are statistically significant (*p* = 0.004). However, the observed changes in the total bilirubin mean values were within the normal range. The statistically significant changes observed in mean values from baseline for white blood cells, platelets, neutrophil counts, lymphocyte counts, eosinophil counts, alanine aminotransferase, total bilirubin, and creatinine were not associated with any signs of toxicity or clinical symptoms.

## 4. Discussion

This study is the first clinical trial to evaluate the safety and tolerability of an antimalarial herbal remedy, Maytenus Senegalese, at ascending doses (400 mg, 600 mg and 800 mg every 8 h for four days) in healthy adult male volunteers. 

There were no deaths or serious adverse events or adverse events considered clinically relevant or resulting in tested product discontinuation or study withdrawal. Furthermore, no solicited or unsolicited clinical adverse events (AEs) were reported in the first 28 days post administration of the tested product. All AEs reported were laboratory adverse events and were graded either 1 or 2 in severity.

All reported adverse events were transient and resolved without any clinical sequelae. The clinical review of physical examination, vital signs, and 12-lead ECG data did not reveal safety signals. The findings of this study are supported by the good pre-clinical safety data of *M. senegalensis*. Preclinical animal studies have shown that orally administered *M. senegalensis* root extracts are well tolerated, without evidence of toxicity or death, in experimental mice [4,15]. 

In this study, there was a significant increase in the mean value of white blood cells and their differentials (neutrophils counts, lymphocyte counts, and eosinophils counts) for the group that received the highest dose of the tested product. This finding suggests the potential immunomodulatory effects of the products tested. WBC and its differences are known for a protective role against foreign bodies and infectious pathogens through the production, transport and distribution of antibodies in the immune response [26]. Plants with immune modulatory effects have been reported to enhance the production of WBC in animals to overcome the stress induced by the plant [27]. This study’s findings are similar to other studies that have reported *M. senegalensis* to be anti-inflammatory [1,3], antimalarial, analgesic [2,4,5], antibacterial and anthelminthic [7,8,9,10,11].

Hemoglobin inside RBC is essential in transferring respiratory gases. It transports oxygen to cells and carbon dioxide to the lungs [28]. The findings of this study revealed that the mean value of red blood cells (RBC) and hemoglobin did not show a significant change from baseline. This finding suggests that the oxygen-carrying capacity of the blood and the amount of oxygen delivered to the tissues were not compromised by the tested product. This study finding suggests that the tested product did not cause destruction of existing RBC or inhibit or stimulate the release of erythropoietin in the kidney. This inhibition or stimulation of erythropoietin release is a humoral regulator of RBC production.

The results of the study showed that statistically significant changes in the mean value of platelets in the group that received the highest dose of the tested product. Platelets are fragments of large bone marrow cells that function to aid blood clotting and initiate the repair of the walls of blood vessels [29]. This finding implies that the tested product did not interfere with blood clotting function. This finding implies that the administration of the tested product did not interfere with the blood clotting function of the body. 

AST and ALT levels are the predominant blood markers of liver injury. In this study, there was an increasing trend in mean values of the ALT parameter for the study group that received the highest dose of the tested product up to day seven after administration, and then it started to return to baseline. This finding implies that the administration of *Maytenus senegalese* induced a transient change in ALT levels in healthy adult male volunteers. This study finding is consistent with the findings of a preclinical animal study, which revealed a transient dose-dependent increase in ALT activity following administration of an 800 mg/kg dose of *M. senegalensis* to male rats [30]. 

The level of creatinine is one of the indicators that play an important role in determining the synthetic and excretory functions of the kidney. A significant increase in serum creatinine after administration of the tested product may be an indication of renal failure, especially with regard to glomerular filtration, which directly affects the rate of clearance of waste substances by the kidney [31]. Furthermore, ingestion of cooked meat and exercise can transiently increase serum creatinine to levels that are interpreted as pathological and can lead to misinterpretation during phase I clinical trials in healthy volunteers. Therefore, it is recommended that healthy volunteers in phase I studies be tested for serum creatinine under fasting conditions [32]. In this study, there were observed statistically significant increases in the mean value of creatinine from the baseline for the study group that received the highest dose of the tested product. This finding suggests that this observed transient change in the mean creatinine value may be due to the effect of the diet during admission to the ward. 

Bilirubin is the breakdown of hemoglobin in red blood cells and is a by-product of hemolysis (RBC destruction). The measurement of bilirubin allows the evaluation of liver function and hemolytic anemia [33]. In this study, there were observed statistically significant decreases in the mean value of total bilirubin from the baseline for the study groups that received the highest dose of the tested product. However, the observed changes in the mean value of total bilirubin were within the normal range. This finding suggests that the tested product did not cause the breakdown of hemoglobin in the RBCs.

The study was limited by the small number of male volunteers enrolled. More research should be done to investigate the effects of gender differences. Nevertheless, the information presented here lays a foundation for future follow-up studies to evaluate the efficacy of *M. senegalensis* as an antiparasitic agent. In addition, there is a lack of pharmacokinetic data for the *M. senegalensis* extract. The availability of pharmacokinetics data for herbal remedy extracts is crucial for the prediction of potential herb–drug interactions and herbal pharmacological efficacy [34]. Pharmacokinetic studies on the *M. senegalensis* extract are needed in the future. However, it would be a challenge due to the chemical complexity [35].

## 5. Conclusions

In summary, an antimalarial herbal remedy, *M. senegalensis*, was demonstrated to be safe, tolerable, and free of any untoward toxic effects in healthy male volunteers when administered at a dose of 800 mg every 8 h for four days. The statistically significant changes in the mean values from baseline for white blood cells, neutrophils, lymphocytes, eosinophils, alanine aminotransferase, total bilirubin, and creatinine were not associated with any toxicity or clinical symptoms. 

The design of this study may be adapted to evaluate other herbal products with medicinal potentials and fast-track finding solutions for malaria and other health problems in Africa. Therefore, the findings of this study contribute to the effort to develop medical evidence and fill the scientific gap created by the lack of safety data on most herbal remedies.

## Figures and Tables

**Table 1:** Compilation of analysis reports.

Product name: *M. senegalensis* Batch number: MS2020-0002 Description: 200 mg Capsule				
01 Product description		Specification		Test method
	Plant part used	Root bark		Visual
	Botanical name	*M. senegalensis*		Macroscopic
	Plant: % of Extract yield	11.2%		By weight
02 Physical data		Specification	Result	Test method
	Appearance	-	Capsule	Visual
	Aroma	Neutral Odor	Odor of Ingredients	Organoleptic test
	Particle Size	-	900 µm	Sieve analysis
	Average Moisture %	-	1.87	Loss on drying method
03 Chemical data		Specification	Result	Test method
	Alkaloids	-	Detected	Precipitation test
	Anthraquinones	-	Not detected	Color test
	Flavonoids	-	Not detected	Color test
	Saponins	-	Detected	Foam test
	Pesticide residues	-	Not detected	“GC-MS/MS”
	Lead	10 ppm	<0.001 ppm	ICP-OES
	Arsenic	2 ppm	<0.005 ppm	ICP-OES
	Cadmium	0.3 ppm	<0.001 ppm	ICP-OES
	Chromium	2 ppm	<0.001 ppm	ICP-OES
04 Microbiological data		Specification	Result	Test method
	Aerobic bacteria	Max 10^5^ (cfu/g)	3.4 × 10^2^ (cfu/g)	Spread technique
	Clostridia	Absent	0 per gram	Spread technique
	Total Coliform counts (MPN/g)	Max 10^3 (^MPN/g)	<3 (MPN/g)	MPN technique
	E. coli count	Absent	<3 (MPN/g)	MPN technique
	Salmonella spp.	Absent	Absent	Conventional technique
	Yeast and Mould Identification: Yeasts	Max 10^4 (^cfu/g)	0 (cfu/g)	Spread technique
	Aflatoxin G1, μg/Kg	-	Not detected	FCL/SOP-TM/13-02
	Aflatoxin G2, μg/Kg	-	Not detected	FCL/SOP-TM/13-02
	Aflatoxin B2, μg/Kg	-	Not detected	FCL/SOP-TM/13-02
	Aflatoxin B1, μg/Kg	-	Not detected	FCL/SOP-TM/13-02
	Total Aflatoxin, μg/Kg	-	Not detected	FCL/SOP-TM/13-02
05 Additional information				
	Extraction method	Cold maceration		
	Packing	Hard-shelled capsule, then in blister bags containing 100 capsules		
	Storage	Temperature below 30 °C away from exposure to sunlight and moisture		
	Shelf life	2 years		
	Country of origin/manufactured	Tanzania		
	Non-irradiation	This material has not been subjected to irradiation		

**Table 2:** Summary of demographic characteristics of each study group.

Characteristics	Statistics	Group 1 (*n* = 3)	Group 2 (*n* = 3)	Group 3 (*n* = 3)	Group 4 (*n* = 3)	All (*n* = 12)
Age	Mean	27.21	23.63	28.62	31.35	27.70
	SD	2.62	4.88	4.95	11.14	6.41
	Range	4.96	9.76	9.43	19.49	25.48
Sex						
Male	*n* (%)	3 (25)	3 (25)	3 (25)	3 (25)	12 (100)
Race						
African	*n* (%)	25	25	25	25	100
Height, Kg	Mean	169.33	164.67	170.5	165.17	167.42
	SD	4.16	3.06	3.91	7.75	5.06
	Range	4.16	6	7	15.5	16.5
Weight, Kg	Mean	76.67	55.67	63.67	60	64
	SD	6.11	6.66	2.89	8.54	9.84
	Range	12	13	5	17	32
BMI, Kg/m^2^	Mean	26.71	20.58	21.91	21.89	22.77
	SD	1.166	2.99	1.03	1.06	2.87
	Range	2.24	5.42	1.8	2.09	9.05

Abbreviations: *n* = number of study volunteers; SD = standard deviation; BMI = body mass index.

**Table 3:** Summary of solicited systemic advents in the first 8 days post treatment.

Characteristics	Group 1 (*n* = 3)	Group 2 (*n* = 3)	Group 3 (*n* = 3)	Group 4 (*n* = 3)
Nervous System				
Drowsiness	0	0	0	0
Nervousness	0	0	0	0
Insomnia	0	0	0	0
Nightmares	0	0	0	0
Shivering	0	0	0	0
Numbness	0	0	0	0
Ageusia	0	0	0	0
Tinnitus	0	0	0	0
Blurred vision	0	0	0	0
Unpleasant taste	0	0	0	0
Thirst	0	0	0	0
Cardiovascular System				
Fast heartbeat	0	0	0	0
Irregular heartbeat	0	0	0	0
Heartbeat awareness	0	0	0	0
Respiratory system				
Cough	0	0	0	0
Chest pain	0	0	0	0
Stuffy nose	0	0	0	0
Difficulty in breathing	0	0	0	0
Gastrointestinal System				
Heartburn	0	0	0	0
Abdominal pain	0	0	0	0
Diarrhea	0	0	0	0
Nausea and vomiting	0	0	0	0
Constipation	0	0	0	0
Intestinal wind	0	0	0	0
Abnormal stool color	0	0	0	0
Genito-urinary System				
Dysuria	0	0	0	0
Polyuria	0	0	0	0
Nocturia	0	0	0	0
Dark urine	0	0	0	0
Change in sexual ability/desire	0	0	0	0
Muco-cutaneous System				
Skin rash	0	0	0	0
Pruritus	0	0	0	0
Dry mouth	0	0	0	0
Others				
Jaundice	0	0	0	0
Fever	0	0	0	0
Headache	0	0	0	0

**Table 4:** Results of the hematology test for the first 28 days of administration of *M. senegalensis* in study groups 1, 2, 3 and 4.

Hematology Parameters Reference Range (Unit)	Visit Code	G1				G2				G3 and 4			
		*n*	Mean ± SD	Median (Range)	* *p* Value	*n*	Mean ± SD	Median (Range)	* *p* Value	*n*	Mean ± SD	Median (Range)	*p* Value
White Blood Cells (WBC) 3.48–9.11 (10^3^/µL)	Day 1	3	5.76 ± 2.39	5.28 (3.65, 8.36)	0.13 *	3	4.097 ± 0.90	3.68 (3.48, 5.13)	0.05 *	6	5.13 ± 1.57	4.99 (3.2, 7.76)	0.003 *
	Day 3	3	4.723 ± 0.55	4.93 (4.1, 5.14)		3	5.347 ± 1.26	5.45 (4.04, 6.55)		6	7.465 ± 2.62	7.09 (4.78, 11.46)	
	Day 7	3	4.87 ± 0.77	4.56 (4.33, 5.77)		3	5.863 ± 2.18	6.17 (3.55, 7.87)		6	7.66 ± 2.30	6.7 (5.85, 11.66)	
	Day 14	3	4.53 ± 0.78	4.17 (4, 5.42)		3	4.313 ± 1.74	4.16 (2.66, 6.12)		6	5.68 ± 1.54	4.91 (3.97, 8.23)	
	Day 28	3	3.883 ± 0.95	3.41 (3.26, 4.98)		3	4.297 ± 1.30	4.95 (2.8, 5.14)		6	5.27 ± 0.94	5.38 (3.68, 6.45)	
Red Blood Cells (RBC) 4.39–6.5 (10^6^/µL)	Day 1	3	5.63 ± 0.37	5.59 (5.28, 6.02)		3	5.28 ± 0.32	5.43 (4.91, 5.5)	0.16 *	6	5.44 ± 0.46	5.26 (5.07, 6.21)	0.07 *
	Day 3	3	5.36 ± 0.37	5.35 (5, 5.74)		3	5.54 ± 0.55	5.78 (4.91, 5.92)		6	5.27 ± 0.46	5.16 (4.82, 6.04)	
	Day 7	3	5.40 ± 0.40	5.45 (4.97, 5.77)	0.07 *	3	5.61 ± 0.37	5.64 (5.23, 5.96)		6	5.34 ± 0.55	5.32 (4.67, 6.1)	
	Day 14	3	5.46 ± 0.39	5.43 (5.09, 5.86)		3	5.34 ± 0.30	5.33 (5.05, 5.64)		6	5.28 ± 0.52	5.26 (4.75, 6.01)	
	Day 28	3	5.36 ± 0.37	5.26 (5.04, 5.77)		3	5.6 ± 0.42	5.47 (5.26, 6.07)		6	5.30 ± 0.42	5.34 (4.82, 5.97)	
Hemoglobin (HGB) 12.6–17.3 (g/dL)	Day 1	3	15.13 ± 1.66	15.3 (13.4, 16.7)	0.08 *	3	13.93 ± 0.46	14.2 (13.4, 14.2)	0.08 *	6	15.33 ± 1.29	15.55 (13.2, 16.8)	0.31 *
	Day 3	3	14.37 ± 1.70	14.5 (12.6, 16)		3	14.73 ± 0.43	14.6 (14.4, 15.2)		6	15.03 ± 1.15	15.15 (13, 16.2)	
	Day 7	3	14.53 ± 1.90	14.6 (12.6, 16.4)		3	14.8 ± 0.2	14.8 (14.6, 15)		6	15.13 ± 1.53	15.55 (12.3, 16.7)	
	Day 14	3	14.67 ± 1.65	14.7 (13, 16.3)		3	14.1 ± 0.78	14.5 (13.2, 14.6)		6	14.98 ± 1.37	15.35 (12.6, 16.4)	
	Day 28	3	14.43 ± 1.52	14.7 (12.8, 15.8)		3	14.77 ± 0.95	15.1 (13.7, 15.5)		6	15.1 ± 1.28	15.5 (12.6, 16.1)	
Platelets (PLT) 107–396.2 (10^3^/µL)	Day 1	3	231.67 ± 42.55	234 (188, 273)	0.05 *	3	246 ± 77.31	215 (189, 334)	0.48 *	6	249 ± 44.21	265.5 (175, 291)	0.82 *
	Day 3	3	213.67 ± 57.01	215 (156, 270)		3	241.33 ± 62.78	220 (192, 312)		6	247.83 ± 60.79	257 (169, 319)	
	Day 7	3	210.33 ± 62.07	220 (144, 267)		3	219.66 ± 55.08	193 (183, 283)		6	249.33 ± 61.05	248.5 (168, 319)	
	Day 14	3	236.66 ± 44.74	246 (188, 276)		3	232.66 ± 68.12	228 (167, 303)		6	246.33 ± 50.23	245 (184, 324)	
	Day 28	3	206.67 ± 33.56	209 (172, 239)		3	221 ± 55.76	201 (178, 284)		6	238.5 ± 43.04	247 (186, 300)	
Neutrophils counts (NEUT_N) 1.18–5.46 (10^3^/µL)	Day 1	3	3.11 ± 2.46	1.97 (1.42, 5.93)	0.07 *	3	1.48 ± 0.32	1.61 (1.12, 1.72)	0.06 *	6	2.77 ± 1.49	2.47 (1.14, 5.46)	0.02 *
	Day 3	3	2.15 ± 0.45	1.93 (1.85, 2.66)		3	2.39 ± 0.56	2.29 (1.88, 2.99)		6	4.23 ± 2.19	3.93 (1.97, 7.54)	
	Day 7	3	2.38 ± 0.20	2.34 (2.2, 2.59)		3	2.2 ± 0.41	2.4 (1.73, 2.47)		6	4.13 ± 1.96	3.52 (1.82, 7.26)	
	Day 14	3	1.82 ± 0.13	1.89 (1.67, 1.9)		3	1.44 ± 0.65	1.64 (0.71, 1.96)		6	2.76 ± 1.16	2.43 (1.77, 4.88)	
	Day 28	3	1.40 ± 0.30	1.28 (1.19, 1.74)		3	1.56 ± 0.72	1.19 (1.1, 2.39)		6	2.645 ± 0.76	2.81 (1.26, 3.41)	
Lymphocytes counts (LYMP_N) 1.19–3.4 (10^3^/µL)	Day 1	3	1.95 ± 0.36	1.74 (1.74, 2.36)		3	1.98 ± 0.35	1.81 (1.75, 2.38)	0.86 *	6	1.85 ± 0.10	1.83 (2.03)	0.001 *
	Day 3	3	1.85 ± 0.34	1.72 (1.6, 2.23)		3	2.01 ± 0.53	1.9 (1.55, 2.59)		6	2.40 ± 0.37	2.30 (2.99)	
	Day 7	3	1.85 ± 0.30	1.79 (1.59, 2.17)	0.25 *	3	2.53 ± 1.20	2.98 (1.17, 3.44)		6	2.61 ± 0.48	2.60 (3.37)	
	Day 14	3	2.02 ± 0.43	1.92 (1.67, 2.48)		3	1.93 ± 0.39	2.02 (1.51, 2.27)		6	1.83 ± 0.27	1.73 (2.24)	
	Day 28	3	1.40 ± 0.30	1.28 (1.19, 1.74)		3	1.94 ± 0.66	2.08 (1.23, 2.52)		6	1.94 ± 0.24	1.91 (2.24)	
Eosinophils counts (EOS_N) 0–0.78 (10^3^/µL)	Day 1	3	0.31 ± 0.24	0.20 (0.15, 0.58)	0.29 *	3	0.28 ± 0.27	0.24 (0.03, 0.56)	0.90 *	6	0.12 ± 0.08	0.11 (0.03, 0.24)	0.01 *
	Day 3	3	0.27 ± 0.23	0.18 (0.11, 0.53)		3	0.38 ± 0.39	0.25 (0.08, 0.82)		6	0.21 ± 0.10	0.24 (0.09, 0.35)	
	Day 7	3	0.26 ± 0.21	0.16 (0.12, 0.5)		3	0.46 ± 0.45	0.25 (0.15, 0.98)		6	0.19 ± 0.10	0.22 (0.05, 0.32)	
	Day 14	3	0.27 ± 0.23	0.21 (0.08, 0.52)		3	0.45 ± 0.57	0.18 (0.07, 1.1)		6	0.18 ± 0.15	0.14 (0.03, 0.47)	
	Day 28	3	0.23 ± 0.16	0.17 (0.12, 0.41)		3	0.25 ± 0.19	0.22 (0.08, 0.45)		6	0.15 ± 0.08	0.17 (0.03, 0.24)	

Data are represented as mean ± SD and median (range) from baseline to day 28. * Friedman test.

**Table 5:** Results of the biochemistry test for the first 28 days of administration of *M. senegalensis* in the study groups 1, 2, 3 and 4.

Biochemistry Parameters Reference Range (Unit)	Visit Code	G1				G2				G3 and 4			
		*n*	Mean ± SD	Median (Range)	* *p* Value	*n*	Mean ± SD	Median (Range)	* *p* Value	*n*	Mean ± SD	Median (Range)	*p* Value
Aspartate aminotransferase (AST) 12.3–74.7 (U/L)	Day 1	3	21.47 ± 6.45	22.3 (16.1, 29)	0.07 *	3	22.5 ± 5.55	22.4 (17, 28.1)	0.14 *	6	28.6 ± 4.67	27.25 (22.4, 36.1)	0.62 *
	Day 3	3	27.47 ± 13.92	24.6 (15.2, 42.6)		3	31.33± 7.36	29.9 (24.8, 39.3)		6	39.42 ± 23.31	34.85 (19.2, 84.1)	
	Day 7	3	32.5 ± 10.11	29.4 (24.3, 43.8)		3	34.8 ± 12.70	37.7 (20.9, 45.8)		6	53.25 ± 47.17	36.3 (29.3, 149.3)	
	Day 14	3	23.6 ± 5.40	23.8 (18.1, 28.9)		3	23.1 ± 3.40	22.4 (20.1, 26.8)		6	38.2 ± 14.55	35.65 (25.7, 65.6)	
	Day 28	3	23.83 ± 4.76	23.4 (19.3, 28.8)		3	52.83 ± 14.86	58.9 (35.9, 63.7)		6	35.42 ± 5.27	35.7 (26.9, 40.8)	
Alanine aminotransferase (ALT) 3.5–46.8 (U/L)	Day 1	3				3	13.77± 5.25	12.9 (9, 19.4)	0.03	6	23.25 ± 6.49	20.85 (17.3, 34	0.002 *
	Day 3	3				3	28.97 ± 5.66	29.9 (22.9, 34.1)		6	36.23 ± 24.01	27.45 (18.2, 81.8)	
	Day 7	3				3	45 ±21.81	50.4 (21, 63.6)		6	68.62 ± 53.51	46.05 (33.8, 174.7)	
	Day 14	3				3	23.73 ± 0.51	27.8 (11.8, 31.6)		6	48.35 ± 23.55	44.65 (25.3, 89.1)	
	Day 28	3				3	12.87 ± 2.82	13.8 (9.7, 15.1)		6	26.87 ± 13.60	21.1 (16.1, 53.3)	
Total Bilirubin 3.1–31.1 (μmol/L)	Day 1	3	11.07 ± 4.38	12.7 (6.1,14.4)	0.23 *	3	8.63± 0.68	8.4 (8.1, 9.4)	0.41	6	15.65 ± 13.35	10.4 (7.7, 42.7)	0.0004 *
	Day 3	3	5.6 ± 2.98	6.1 (2.4, 8.3)		3	7.43 ± 2.40	8.4 (4.7, 9.2)		6	08.03 ± 6.91	5.2 (2.9, 21.6)	
	Day 7	3	6.37 ± 2.88	6.8 (3.3, 9)		3	8.93± 2.90	9 (6, 11.8)		6	09.4 ± 6.39	6.3 (4.9, 21.7)	
	Day 14	3	8.13 ± 1.83	7.5 (6.7,10.2)		3	11.13 ± 4.85	8.9 (7.8, 16.7)		6	15.27 ± 9.09	13.2 (7.2, 32.9)	
	Day 28	3	8.67± 5.55	8.5 (3.2, 14.3)		3	9.93 ± 1.86	9.7 (8.2, 11.9)		6	13.37 ± 8.69	10.25 (7.2, 30.2)	
Creatinine 49–95.3 (μmol/L)	Day 1	3	83.33 ± 7.77	81 (77, 92)	0.10 *	3	77.67 ± 7.57	81 (69, 83)	0.13	6	79.67 ± 8.45	82.5 (67, 88)	0.03 *
	Day 3	3	70± 3	70 (67, 73)		3	76.67± 7.57	80 (68, 82)		6	96.83 ± 9.70	99 (82, 108)	
	Day 7	3	86 ± 5.57	87 (80, 91)		3	82 ±10.54	83 (71, 92)		6	80.27 ± 8.31	79.8 (7, 91)	
	Day 14	3	87.33± 2.08	88 (85, 89)		3	79.67 ± 8.02	79 (72, 88)		6	83.13 ± 7.02	83 (75.3, 94)	
	Day 28	3	89 ± 3.61	90 (85, 92)		3	85.33 ± 7.37	88 (77, 91)		6	88.03 ± 10.75	88.4 (70.8, 104.3)	

Data are represented as mean ± SD and median (range) from baseline to day 28. * Friedman test.

## Data Availability

For reasons of protection of study volunteers, the Tanzania National Health Research Ethics Review Committee, National Institute for Medical Research (NIMR), restricts access to data. In addition, sensitive data was collected from clinical trial study volunteers who did not consent to open data access. However, criteria-eligible researchers with an interest in the data can request anonymized data access through the Chairperson of the National Health Research Ethics Review Committee. The contact information for the National Health Research Ethics Review Committee to which data requests may be sent is: National Institute for Medical Research (NIMR), 2448 Ocean Road, P.O. Box 9653, Dar es Salaam, Tanzania; Telephone: +255 22 212140. Email: ethics@nimr.or.tz.
